# Editorial Note: Cumulative burden of non-communicable diseases predicts COVID hospitalization among people with HIV: A one-year retrospective cohort study

**DOI:** 10.1371/journal.pone.0346212

**Published:** 2026-04-01

**Authors:** The PLOS One Editors

The *PLOS One* Editors issue this Editorial Note for this article [1] due to concerns related to the peer review process. Readers are encouraged to approach the article [1] with careful consideration.

The Editors have no evidence of any author involvement in the peer review concerns.
